# Carabid beetle diversity and distribution in Boston Harbor Islands national park area (Coleoptera, Carabidae)

**DOI:** 10.3897/zookeys.147.2111

**Published:** 2011-11-16

**Authors:** Robert L. Davidson, Jessica Rykken, Brian Farrell

**Affiliations:** 1 Section of Invertebrate Zoology, Carnegie Museum of Natural History, 4400 Forbes Avenue, Pittsburgh PA 15213; 2 Museum of Comparative Zoology, Harvard University, 26 Oxford Street, Cambridge, MA 02138

**Keywords:** Carabidae, Boston Harbor Islands, biodiversity inventory, introduced species, state records Massachusetts, New York, Pennsylvania, country record U. S.

## Abstract

As part of an All Taxa Biodiversity Inventory in Boston Harbor Islands national park area, an inventory of carabid beetles on 13 islands was conducted. Intensive sampling on ten of the islands, using an assortment of passive traps and limited hand collecting, resulted in the capture of 6,194 specimens, comprising 128 species. Among these species were seven new state records for Massachusetts (*Acupalpus nanellus,* *Amara aulica,* *Amara bifrons*, *Apenes lucidulus*, *Bradycellus tantillus*, *Harpalus rubripes* and *Laemostenus terricola terricola*—the last also a new country record; in passing we report also new state records for *Harpalus rubripes* from New York and Pennsylvania, *Amara ovata* from Pennsylvania, and the first mainland New York records for *Asaphidion curtum*). For most islands, there was a clear relationship between species richness and island area. Two islands, however, Calf and Grape, had far more species than their relatively small size would predict. Freshwater marshes on these islands, along with a suite of hygrophilous species, suggested that habitat diversity plays an important role in island species richness. Introduced species (18) comprised 14.0% of the total observed species richness, compared to 5.5% (17 out of 306 species) documented for Rhode Island. We surmise that the higher proportion of introduced species on the islands is, in part, due to a higher proportion of disturbed and open habitats as well as high rates of human traffic. We predict that more active sampling in specialized habitats would bring the total carabid fauna of the Boston Harbor Islands closer to that of Rhode Island or eastern Massachusetts in richness and composition; however, isolation, human disturbance and traffic, and limited habitat diversity all contribute to reducing the species pool on the islands relative to that on the mainland.

## Introduction

The Boston Harbor Islands national park area comprises 34 islands and peninsulas lying within 20 km of downtown Boston, Massachusetts, U.S.A. (Fig. 1). The islands have a long history of use and colonization by both Native and European Americans. Island landscapes have been altered over time with fishing settlements and agriculture, military forts and other institutional buildings, a landfill and sewage treatment plants, and by many other activities (Kales 2007). Over the last forty years, several of the islands and peninsulas have become state parks, private conservation lands, and outdoor classrooms for environmental education programs. In 1996, congress designated 34 islands and peninsulas in the harbor as a “national park area”, to be managed in partnership with the National Park Service and eleven other stakeholders. A primary purpose and mission of the park is “to preserve and protect a drumlin island system within Boston Harbor, along with associated natural, cultural, and historic resources” (National Park Service 2002). As is the case for most national parks, however, relatively little is known about the natural resources which park managers are mandated to protect. While surveys for vascular plants and most vertebrates are ongoing, the largest component of biological diversity, the invertebrate fauna, has received little attention (with the exception of Lepidoptera; Mello 2005).

**Figure:**
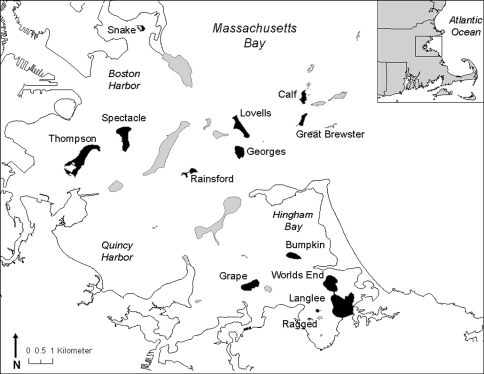
**Figure 1.** Location of Boston Harbor Islands national park area. Islands/peninsulas sampled for carabid beetles are shaded in black.

In an effort to learn more about its natural resources, the park initiated an All Taxa Biodiversity Inventory (ATBI) in 2005, with a primary objective cataloguing arthropod biodiversity across the islands. The ATBI concept, initially conceived by Janzen and Hallwachs (1994), has as its ultimate goal the documentation of all species occurring within the boundaries of a natural area (such as a park) in a relatively short period of time. This comprehensive and efficient approach to cataloguing diversity came in response to ever-increasing levels of species and habitat loss in the tropics and elsewhere, and the realization that fundamental knowledge about existing species diversity in even the smallest reserves is lacking. Currently, ATBI efforts are underway in known hotspots of biodiversity such as the Dominican Republic (Farrell 2005) and the Great Smoky Mountain National Park (Nichols and Langdon 2007). The Boston Harbor Islands ATBI applies the same approach in an urban island landscape, where biodiversity is expected to be comparatively low due to bioregion, high levels of human disturbance, limited area, and isolation. However, an ATBI in this landscape is also expected to provide novel information about many arthropod taxa, including patterns of island colonization on a small spatial scale, species resilience to human disturbance, and the relative proportion of introduced species in an historically active harbor compared with the adjacent mainland.

Carabid beetles are a focal group of the ATBI because they are one of the most diverse and abundant beetle families on the islands, both in species and individuals; because they are relatively well known taxonomically and distributionally; and because there is expertise available for their identification. There is also enough information about them from the adjacent mainland to make cautious comparison possible, though unfortunately nothing as up to date as the current ATBI. Sikes (2004) records 306 carabid species from Rhode Island. Bousquet and Larochelle (1993) record 424 species from Massachusetts (and another dozen or so will need to be added to accommodate species either newly described or split from existing species since their catalogues). A high percentage of these will occur in the eastern half of the state on the mainland adjacent to the islands.

The objectives of this study are to: (1) inventory the carabid beetle fauna of a subset of islands (and one peninsula) in the park; (2) document their patterns of distribution across islands; (3) assess the regional significance of species occurrences in Boston Harbor and (4) compare the carabid fauna on the islands with that on the mainland of Rhode Island, including similarity of species composition, species richness, and the proportion of introduced species. We use the words introduced and introduction in this paper to refer to invasive species which arrived accidentally or incidentally, e.g., in ballast through human commerce, as opposed to deliberate introductions, e.g., as biological control agents.

## Methods

**Site description.** The islands and one peninsula (World’s End) sampled for carabid beetles range in size from 1.1 to 104.5 ha (Table 1). The majority of the islands are drumlins, formed by deposits of glacial till in Boston basin; a few (Ragged, Langlee, Calf) are bedrock outcrops (National Park Service 2002). Sea level rise associated with glacial melting 15,000 years ago flooded the basin and isolated the islands, and they now lie between 0.3 and 3.3 km from the nearest mainland. Most of the intertidal areas on the islands are mixed coarse substrate such as gravel and cobble, but a few islands have sandy beaches, and some have areas of bedrock shoreline (Bell et al. 2005). The dominant vegetation communities on most islands include forest, woodland, maritime shrub, old field, and beach strand (Elliman 2005). Non-native woody and herbaceous plant species dominate many of the vegetation communities on the islands (44% of all plant species on the islands), but the dominant shrub on almost all of the islands is native staghorn sumac (*Rhus typhina*). Spectacle Island is a reclaimed landfill that was replanted within the last decade and is currently mostly open habitat. Salt marshes and brackish marshes occur on several of the islands, but freshwater is extremely scarce.

Almost all of the islands in the park are open to human visitors. Several islands (Bumpkin, Georges, Grape, Lovells, Spectacle, and Thompson) are serviced by public ferries between May and October. Portions of these islands and World’s End are also actively landscaped (e.g., mowing, clearing brush). Islands with ferry service and World's End receive hundreds to tens of thousands of visitors per year (Table 1), with Spectacle and Georges Islands serving as hubs in the ferry system. Thompson Island, an education center, also receives very high numbers of visitors. The remaining islands, which do not have public ferry service, probably receive on the order of hundreds of visitors per year, but records are not available.

**Field sampling design**. Islands were sampled with varying intensities and in different years (Table 1). Three islands (Thompson, Grape, and Langlee) were sampled between August and October, 2005, in a short pilot season to test field sampling methods. A full-season (May through October) structured sampling design was implemented on Grape and Thompson in subsequent years, and also on seven other islands (Table 1). Three additional islands (Georges, Lovells, and Rainsford) were visited sporadically for hand-collecting only.

On islands with structured sampling, a variety of traps and methods was used to sample different habitats. Sampling was stratified by dominant habitat types: forest, shrubland, meadow, beach (above the high tide line), salt- or brackish marsh, freshwater marsh or pond edge. Larger islands had more sampling sites than smaller islands, but had fewer samples per unit area overall. Sampling methods included: pitfall traps (plastic cups dug into the ground, 90 mm diameter at the mouth) in groups of three traps per site; litter samples run through Berlese funnels; malaise traps, one per island, rotated through different habitats over the season; mercury vapor and UV lights; and bowl traps laid out every 5 m in transects of 70 m, placed near flowering plants in open areas. Sampling generally occurred every other week, during which pitfall and malaise traps were open for the full week; light traps were run one night in different locations on each island; and bowls were set up and opened for several hours on one sunny day. In addition to the structured passive sampling, active sampling (e.g., hand-collecting, sweep nets, beating sheets) occurred on all visits to the islands. Thompson Island, an outdoor education center, contributed many specimens via hand collections and pitfall samples from student programs.

Carabid beetles were identified to species in part using (Lindroth (1961, 1963, 1966, 1968, 1969a, 1969b), with verification or correction later by Davidson to incorporate more recent literature and to bring the nomenclature up to date. Primary sources are too numerous to mention, but some nomenclature and much distributional information was updated with Bousquet and Larochelle (1993), Ball and Bousquet (2001) and Bousquet (2010). All specimens are deposited in the collections of the Museum of Comparative Zoology, Harvard University.

**Data analysis.** To estimate the absolute (versus observed) number of species on the islands sampled, we used the Chao 1 species richness estimator (Chao 1984; Colwell and Coddington 1994), which is based on the number of rare species (singletons and doubletons) in a sample:

S_Chao 1_ = S_obs_ + F_1_^2^/2F_2_

where S_obs_ is the number of observed species in the sample, and F_1 _and F_2_ are the number of observed species represented by one and two individual(s), respectively. We used the program EstimateS 8.2 for these calculations (Colwell 2009).

**Table 1.** Area, isolation, human visitation rates, and sampling design for each island sampled in Boston Harbor Island national park area.

Island	Code	Terrestrial	Isolation	Human	Year(s)	Sampling effort: # sites (# samples)				
		area (ha)	(km)	visitation3	sampled4	Pitfall	Litter	Malaise	Light	Bowl
Bumpkin¹	BM	12.2	627	933	2006	5 (21)	0	3 (5)	3 (3)	0
Calf	CF	7.5	3268	NA	2007	5 (39)	7 (9)	2 (6)	1 (1)	4 (7)
Georges	GE	15.8	1453	67,655		0	0	0	1 (1)	0
Grape	GP	21.9	456	808	2005, 2008	13 (55)	12 (30)	8 (9)	4 (6)	19 (29)
Great Brewster	GB	7.5	2339	NA	2006	6 (45)	11 (15)	3 (7)	2 (2)	2 (2)
Langlee	LN	1.8	492	NA	2005	5 (25)	5 (20)	3 (10)	5 (20)	0
Lovells	LV	19.6	2177	5,576		0	1 (1)	0	3 (3)	7 (7)
Rainsford	RF	6.6	2390	NA		0	0	0	0	0
Ragged	RG	1.1	320	NA	2006	5 (31)	12 (20)	2 (6)	1 (2)	0
Snake	SN	2.9	344	NA	2007	4 (20)	4 (6)	2 (4)	0	2 (2)
Spectacle	SP	34.6	1907	35,441	2007	5 (39)	3 (12)	4 (7)	1 (1)	12 (12)
Thompson¹	TH	54.2	517	17,621	2005, 2007	31 (119)	16 (36)	10 (27)	13 (8)	13 (15)
World’s End²	WE	104.5	0	49,664	2006	9 (63)	13 (21)	7 (13)	8 (7)	16 (22)

^1^ Island connected to mainland at very low tides

^2^ Peninsula, connected to mainland at all times

^3^ Visitor counts for 2007. Counts for all islands represent ferry passengers only (visitors in private boats not included), and count for WE represents drive-up visitors. NA = no available data/no ferry service. (National Park Service 2010, Boston Harbor Island visitor statistics, unpublished report).

^4^ Years for structured sampling, does not include all hand-collecting events. 2005 sampled Aug-Oct only.

## Results

We collected a total of 6,194 carabid specimens, comprising 128 species (Table 2). Seven species were recorded from Massachusetts for the first time, and one of these, *Laemostenus terricola*, was also a new record for the U.S. (see accounts below). The six most abundant species (*Harpalus rufipes*, *Amara bifrons*, *Pterostichus mutus*, *Carabus nemoralis*, *Poecilus lucublandus* and *Synuchus impunctatus*) made up 59.4% of the total catch. Thirty-five species were represented by a single specimen, and 67 species (over half the total) were represented by five or fewer specimens. The high proportion of singletons and doubletons contributed to an estimated absolute species richness of 189 species (95% CI: 155, 269). Introduced species (18) comprised 14.0% of the total observed species richness, and 45.5% of total specimen abundance. Three of the six most abundant carabid species overall were introduced.

The distribution of carabids across islands varied greatly by species. The most widespread species, the abundant *Poecilus lucublandus*, was collected on ten islands. However, there was no clear relationship between total abundance of a species and its distribution across multiple islands. For instance, *Amara bifrons* was the second most abundant species overall (822) but occurred on only 3 islands, with 815 individuals on Spectacle Island alone. In contrast, we collected *Harpalus rubripes* on six islands, but the total catch comprised only ten specimens. Six of the introduced species each occurred on six or more islands.

Among islands that were sampled intensively for at least one full season (Table 1), species richness varied between 16 (Ragged Island) and 63 species (Grape Island). While there was a clear relationship between island area and species richness for seven of the intensively sampled islands (Fig. 2), Grape Island and Calf Island were obvious outliers as each had many more species than expected for its size.

**Table 2.** Carabid specimens collected on islands in Boston Harbor Islands national park area 2005-2009. See Table 1 for island abbreviations.

															Total	
Species	Status†	BM	CF	GE	GP	GB	LN	LV	RG	RF	SN	SP	TH	WE	Spec.	Islands
*Acupalpus (Acupalpus) carus* (LeConte)					1										1	1
*Acupalpus (Acupalpus) hydropicus (LeConte)					1									3	4	2
*Acupalpus (Acupalpus) nanellus Casey	S		1		4				1						6	3
*Acupalpus (Tachistodes) partiarius* (Say)					1		1								2	2
*Acupalpus (Acupalpus) pumilus* Lindroth					1								1		2	2
*Acupalpus (Philodes) rectangulus Chaudoir														1	1	1
*Agonum (Olisares) aeruginosum* Dejean														1	1	1
*Agonum (Olisares) decorum* (Say)			2		1										3	2
*Agonum (Olisares) ferreum Haldeman			1											2	3	2
*Agonum (Olisares) fidele* Casey					15										15	1
*Agonum (Europhilus) gratiosum* (Mannerheim)			31		11	3									45	3
*Agonum (Olisares) melanarium* Dejean			57		187									8	252	3
*Agonum (Agonum) muelleri* (Herbst)	I		4			9							2	2	17	4
*Agonum (Europhilus) palustre* Goulet			2		9										11	2
*Agonum (Olisares) punctiforme (Say)			5												5	1
*Agonum (Europhilus) retractum* LeConte											1				1	1
*Agonum (Olisares) tenue* (LeConte)					1									1	2	2
*Agonum (Europhilus) thoreyi* Dejean														1	1	1
*Amara (Amara) aenea* (DeGeer)	I	1			1										2	2
*Amara (Zezea) angustata* (Say)														1	1	1
*Amara (Bradytus) apricaria* (Paykull)	I		1										1		2	2
*Amara (Curtonotus) aulica (Panzer)	I, S		1		6	7					22	30	15		81	6
*Amara (Bradytus) avida* (Say)												6			6	1
*Amara (Celia) bifrons (Gyllenhal)	I, S		3									815	4		822	3
*Amara (Amara) cupreolata* Putzeys												2		1	3	2
*Amara (Bradytus) exarata Dejean					30	1						49	7	9	96	5
*Amara (Amara) familiaris* (Duftschmid)	I													1	1	1
*Amara (Amara) littoralis* Mannerheim														1	1	1
*Amara (Amara) lunicollis* Schiødte		3				21						3		3	30	4
*Amara (Celia) musculis* (Say)		1	3		14	1						8	20		47	6
*Amara (Amara) ovata (Fabricius)	I		1		3										4	2
*Amara (Paracelia) quenseli* (Schönherr)			1		13			1				2	57		74	5
*Amara (Celia) rubrica* Haldeman					16								4		20	2
*Amphasia (Pseudamphasia) sericea* (T.W. Harris)		1				2							4	2	9	4
*Anisodactylus (Anisodactylus) harrisii* LeConte		5	13		5	6			1		1	19	7	22	79	9
*Anisodactylus (Anisodactylus) nigerrimus* (Dejean)														1	1	1
*Anisodactylus (Anisodactylus) nigrita* Dejean			1												1	1
*Anisodactylus (Gynandrotarsus) rusticus* (Say)		1			4			1					3		9	4
*Apenes lucidulus (Dejean)	S	1												1	2	2
*Apristus latens* (LeConte)			2	30	1			10		1		2	60		106	7
*Apristus subsulcatus* (Dejean)				8	33			3	3	2			247		296	6
*Asaphidion curtum (Heyden)	I												2		2	1
*Atranus pubescens* (Dejean)					1										1	1
*Axinopalpus biplagiatus* (Dejean)			1							1	2				4	3
*Badister (Badister) notatus* Haldeman						1			1				1	1	4	4
*Badister (Trimorphus) transversus* Casey												1			1	1
*Bembidion (Ochthedromus) americanum* Dejean												1			1	1
*Bembidion (Notaphus) constrictum* (LeConte)			1											10	11	2
*Bembidion (Notaphus) contractum* Say													4		4	1
*Bembidion (Trepanedoris) frontale* (LeConte)			1		18									4	23	3
*Bembidion (Eupetedromus) graciliforme* Hayward			1		9									2	12	3
*Bembidion (Lymnaeum) nigropiceum (Marsham)	I				1					9			72		82	3
*Brachinus (Neobrachinus) vulcanoides Erwin														38	38	1
*Bradycellus (Stenocellus) rupestris* (Say)					1	1						3			5	3
*Bradycellus (Stenocellus) tantillus (Dejean)	S											1			1	1
*Calathus (Neocalathus) opaculus* LeConte					2						3	6	3		14	4
*Carabus (Archicarabus) nemoralis* O. F. Müller	I	3	13		69	174					14		187	23	483	7
*Chlaenius (Anomoglossus) emarginatus* Say		1			3	1					10		5	6	26	6
*Chlaenius (Chlaeniellus) impunctifrons* Say					7										7	1
*Chlaenius (Chlaeniellus) pennsylvanicus pennsylvanicus* Say			21												21	1
*Chlaenius (Chlaeniellus) tricolor tricolor* Dejean					1						3	1	1	3	9	5
*Cicindela (Cicindela) sexguttata* Fabricius														1	1	1
*Clivina (Clivina) fossor* (Linnaeus)	I		5												5	1
*Colliuris (Cosnania) pensylvanica* (Linnaeus)		1													1	1
*Cymindis (Cymindis) americana Dejean							1								1	1
*Cymindis (Pinocodera) limbata* Dejean							1		1				2	3	7	4
*Cymindis (Cymindis) neglecta* Haldeman					2										2	1
*Cymindis (Cymindis) pilosa* Say													1		1	1
*Cymindis (Pinacodera) platicollis (Say)		4			1		1						2	5	13	5
*Dicaelus (Paradicaelus) dilatatus dilatatus* Say														1	1	1
*Dicaelus (Paradicaelus) elongatus* Bonelli		13			7		4		26					23	73	5
*Dicaelus (Paradicaelus) politus Dejean									1					2	3	2
*Diplocheila (Isorembus) obtusa* (LeConte)					2	2					1	1	1		7	5
*Dromius (Dromius) piceus* Dejean														1	1	1
*Dyschirius (Dyschiriodes) dejeanii* Putzeys (=integer LeConte)			2												2	1
*Dyschirius (Dyschiriodes) globulosus* (Say)									5		1		3		9	3
*Dyschirius (Dyschiriodes) setosus* LeConte						1									1	1
*Elaphropus incurvus* (Say)			23										3	1	27	3
*Elaphropus vernicatus* (Casey)						5									5	1
*Elaphropus xanthopus* (Dejean)			6									1			7	2
*Harpalus (Harpalus) affinis* (Schrank)	I		10			1					3		16	2	32	5
*Harpalus (Megapangus) caliginosus* (Fabricius)					1										1	1
*Harpalus (Pseudoophonus) compar* LeConte					1										1	1
*Harpalus (Pseudoophonus) erythropus* Dejean			1										1	2	4	3
*Harpalus (Harpalus) opacipennis* (Haldeman)			4			1						33	1		39	4
Harpalus (Pseudoophonus) pensylvanicus** (DeGeer)		43	2		12	3			2		1		19	6	88	8
*Harpalus (Harpalus) rubripes (Duftschmid)	I,S				1	1					3	1	3	1	10	6
*Harpalus (Pseudoophonus) rufipes* (DeGeer)	I	5	95		184	82					38	537	12	57	1010	8
*Harpalus (Harpalus) somnulentus* Dejean						1									1	1
*Laemostenus (Pristonychus) terricola terricola (Herbst)	I, C				3										3	1
*Lebia (Lebia) analis Dejean								1							1	1
*Lebia (Loxopeza) grandis Hentz								1							1	1
*Lebia (Lebia) pumila* Dejean											1				1	1
*Lebia (Lebia) solea* Hentz													2	2	4	2
*Lebia (Lebia) viridipennis Dejean														1	1	1
*Lebia (Lebia) viridis* Say									1			1			2	2
*Notiobia (Anisotarsus) terminata* (Say)					3							2	1	2	8	4
*Notiophilus aeneus* (Herbst)					5									11	16	2
*Ophonus (Metophonus) puncticeps* Stephens	I	2			1	2						1	39	6	51	6
*Oxypselaphus pusillus* (LeConte)		2			18	1								2	23	4
*Patrobus (Neopatrobus) longicornis* (Say)			1		7										8	2
*Perigona (Perigona) nigriceps* (Dejean)	I						2								2	1
*Platynus (Platynus) decentis* (Say)					56				2					1	59	3
*Poecilus (Poecilus) chalcites* (Say)					1										1	1
*Poecilus (Poecilus) lucublandus* (Say)		42	22		126	19	1	1	6			11	11	92	331	10
*Pterostichus (Lamenius) caudicalis* (Say)			6		15										21	2
*Pterostichus (Melanius) corvinus* (Dejean)					50										50	1
*Pterostichus (Phonias) femoralis* (Kirby)					1										1	1
*Pterostichus (Pseudomaseus) luctuosus* (Dejean)			7		70									1	78	3
*Pterostichus (Morphnosoma) melanarius* (Illiger)	I		6		92		9	4			3	61	3		178	7
*Pterostichus (Bothriopterus) mutus* (Say)		10	2		212	12	34		50			1	321	82	724	9
*Pterostichus (Phonias) patruelis* (Dejean)			26												26	1
*Pterostichus (Bothriopterus) pensylvanicus* LeConte		5			11		2		5				7	3	33	6
*Pterostichus (Hypherpes) tristis* (Dejean)													1	9	10	2
*Scarites (Scarites) subterraneus Fabricius						1									1	1
*Selenophorus hylacis (Say)												2			2	1
*Selenophorus opalinus* (LeConte)		1									1				2	2
*Sphaeroderus stenostomus lecontei* Dejean														3	3	1
*Stenolophus (Agonoderus) comma* (Fabricius)									1				3		4	2
*Stenolophus (Agonoleptus) conjunctus* (Say)		1			5								1		7	3
*Stenolophus (Agonoderus) lineola* (Fabricius)			4										2		6	2
*Stenolophus (Stenolophus) ochropezus* (Say)		2	6		9			6				5	8	9	45	7
*Stenolophus (Agonoleptus) rotundicollis* (Haldeman)					1										1	1
*Syntomus americanus* (Dejean)		1			3								1		5	3
*Synuchus (Pristodactyla) impunctatus* (Say)		4			46	7	184						49	24	314	6
*Tachyta angulata* Casey														1	1	1
*Trichotichnus (Trichotichnus) autumnalis (Say)									1						1	1
*Xestonotus lugubris* (Dejean)					2										2	1
Total no. specimens per island		153	395	38	1418	366	240	28	107	13	108	1606	1220	502		
Total no. species per island		24	41	2	63	27	11	9	16	4	17	29	48	55		

* Species of special interest, individual species accounts in text.

^† ^Status: I = introduced; S = state record; C = country recordTable 2. Carabid specimens collected on islands in Boston Harbor Islands national park area 2005-2009. See Table 1 for island abbreviations.

### Accounts of species of special interest

Remarks on habitat and biology are based on the personal experience of one of the authors (Davidson) and the very fine natural history of North American ground beetles by Larochelle and Larivière (2003). Status as state and country records is based largely on the catalogue by Bousquet and Larochelle (1993), updated with subsequent literature as far as known to Davidson.

#### Acupalpus (Acupalpus) hydropicus

(LeConte)

http://species-id.net/wiki/Acupalpus_hydropicus

##### Remarks.

Specimens from New Hampshire and Massachusetts seem to be the most eastern and northern records for this species to date. Bousquet (2010) records it from Vermont and New Hampshire, but not Maine (also Majka et al. 2011), and it is not recorded from Canada. The species occurs in a variety of wet places and is, as far as known, incapable of flight. The species was not at all abundant or widespread, and our records are based on three specimens from one island and World’s End.

#### Acupalpus (Acupalpus) nanellus

Casey, State record

http://species-id.net/wiki/Acupalpus_nanellus

##### Remarks.

This is a State Record for Massachusetts, though not a surprising one as the species is now known from all New England states (recorded from Rhode Island in Sikes 2004 and Maine in Majka et al. 2011), and is also known from Ontario, Québec, New Brunswick and Nova Scotia (Bousquet 2010). The species occurs in a variety of organically rich wet places and is wing dimorphic. Our record is based on six specimens taken on three islands.

#### Acupalpus (Philodes) rectangulus

Chaudoir

http://species-id.net/wiki/Acupalpus_rectangulus

##### Remarks.

Massachusetts specimens seem to represent the eastern end of the range of this species as far as now known, the nearest localities being in Vermont and Québec. It is not recorded from New Hampshire, Maine or northeastern Canada (Bousquet 2010; Majka et al. 2011). The species occurs in a variety of wet places and is fully winged, capable of flight as it has been taken at lights. A single specimen was taken.

#### Agonum (Olisares) ferreum

Haldeman

http://species-id.net/wiki/Agonum_ferreum

##### Remarks.

Specimens from New Hampshire and Massachusetts seem to be the most eastern records for this species to date. It is not recorded from Maine and is not known from Canada east of Ontario (Bousquet 2010; Majka et al. 2011). The species occurs in a variety of organically rich wet places and is fully winged, presumably capable of flight. Three specimens were taken on one island and World’s End.

#### Agonum (Olisares) punctiforme

(Say)

http://species-id.net/wiki/Agonum_punctiforme

##### Remarks.

Specimens from New Hampshire and Massachusetts seem to be the most eastern records for this species to date. It is not recorded from Maine and is not known from Canada east of Ontario (Bousquet 2010; Majka et al. 2011). This species, which is widespread and often very abundant, was surprisingly rare and limited in the survey. It occurs in a very wide variety of wet places and is fully winged, capable of flight as it is taken frequently at lights. Only five specimens were taken, all from one island.

#### Amara (Curtonotus) aulica

(Panzer), State record, Invasive

http://species-id.net/wiki/Amara_aulica

##### Remarks.

This introduced species is a State Record for Massachusetts. It appears to have been introduced in North America sometime before 1929 (Bousquet 2010), presumably in northeast Canada where it has spread as far as Québec. In the United States, it had been recorded previously only from New Hampshire (Bousquet and Larochelle 1993) and Maine (Bousquet 2010; also Majka et al. 2011), so Massachusetts is at present the southern limit of its known range (in North America). It is not recorded from New Hampshire in Bousquet (2010), but we do not know whether this is merely an error or a deliberate correction. The species occurs on relatively dry ground in a variety of open habitats, especially near towns and ports, and is often abundant in seashore drift (Larochelle and Larivière 2003). It is fully winged and taken frequently at lights. It is abundant and widespread on the harbor islands. Our record is based on 81 specimens from six islands. This suggests this species has been in coastal Massachusetts for some time.

#### Amara (Celia) bifrons

(Gyllenhal), State record, Invasive

http://species-id.net/wiki/Amara_bifrons

##### Remarks.

Much like the previous species (*Amara aulica*), this introduced species is a State Record for Massachusetts, also presumably introduced in northeast Canada before 1929 (Bousquet 2010) and spread as far as Québec. It was recorded previously in the United States only from Maine and New Hampshire (Bousquet and Larochelle 1993; Bousquet 2010; Majka et al. 2011), so Massachusetts is currently the southern known limit of its range (in North America). The species occurs in a variety of open areas on dry, sandy, sparsely vegetated ground, and is heavily favored by human disturbance. It is fully winged, is taken frequently at lights, and is often abundant in seashore drift (Larochelle and Larivière 2003). The species was VERY abundant on Spectacle Island, where 801 specimens were taken (see Discussion), and seven specimens were collected on two other islands.

#### Amara (Bradytus) exarata

Dejean

http://species-id.net/wiki/Amara_exarata

##### Remarks.

This is another species which seems to reach its eastern and northern limits in Massachusetts and New Hampshire. It is not yet recorded from Vermont or Maine (Bousquet 2010; Majka et al. 2011), and is known in Canada only from Ontario (Bousquet and Larochelle 1993). It occurs on dry sandy soil in a variety of open areas, and is heavily favored by human disturbance. It is fully winged and a known flier, and it is taken frequently at lights. The species was relatively abundant and widespread, with 96 specimens taken on four islands and World’s End.

#### Amara (Amara) ovata

(Fabricius), Invasive

http://species-id.net/wiki/Amara_ovata

##### Remarks.

Like many European carabids, this species has been spreading from introduction points in both the northeast and the northwest. Bousquet and Larochelle (1993) report it in the west from British Columbia and Alberta, and in the east from Québec, Ontario, New York, Connecticut and Rhode Island. Hieke (2000) adds records from Oregon, Iowa, Virginia, New Hampshire, and the first Massachusetts records (with labels dated 1933 and 1925, which seems to be the earliest known record according to Bousquet (2010)). Bousquet (2010) also adds further records from northeast Canada (New Brunswick, Nova Scotia and Prince Edward Island). Davidson has records from a couple of sites in the eastern half of Pennsylvania (NEW STATE RECORD for Pennsylvania), about a hundred specimens at the Carnegie Museum of Natural History (CMNH) in Pittsburgh. The species occurs on sparsely vegetated dry soil in a variety of open habitats, is favored by human disturbance, is fully winged, and is found frequently in seashore drift (which means it is presumably capable of flight). The survey took five specimens on two islands.

#### Apenes lucidulus

(Dejean), State record

http://species-id.net/wiki/Apenes_lucidulus

##### Remarks.

This is a State Record for Massachusetts and the northeast limit of the known range of this species. It is recorded from as far north and east as New York, Connecticut, Rhode Island and now Massachusetts, but it is not known from Vermont, New Hampshire, Maine or Canada (Bousquet 2010; Majka et al. 2011). The species occurs on moist soil in deciduous woodland, often under leaf litter. It is wing dimorphic, but fully winged individuals are taken frequently at lights and in seashore drift, so the species is certainly capable of flight. A single fully winged specimen was taken at World’s End.

#### Asaphidion curtum

(Heyden), Invasive

http://species-id.net/wiki/Asaphidion_curtum

##### Remarks.

This introduced species was reported from North America first by Cooper (1930) from Long Island, New York. It was not recorded again for fifty years, until Davidson and Langworthy (1980) rediscovered the species on Long Island and found it to be abundant and widespread. Krinsky saw this paper and wrote to Davidson, realizing that this was the species he had from Connecticut (Krinsky 1981), the first mainland records for this taxon. There are no published records of specimens from Massachusetts from the span 1981 to 1989, but the species presumably worked its way through the state as Bell (1989) reported it from New Hampshire. Bousquet and Larochelle (1993) added records from Massachusetts and Maine, and it has since been recorded from Rhode Island (Sikes 2004). This species is now known from all New England states except Vermont, and all records to date are from counties close to the Atlantic coast. Survey work in Columbia County, New York, in 2008, turned up many specimens (Conrad Vispo, Hawthorne Valley Farms Farmscape Ecology Program, brought specimens to Davidson to identify in 2008; Davidson and Acciavatti collected several more in April, 2009; specimens at CMNH). These are the first mainland records for New York, close to the east bank of the Hudson River, and about as far inland as the species has been found so far. It is not as yet known to have crossed the Hudson River or moved south into New Jersey, possibly reflecting lack of sampling effort. The species occurs on moist, bare or sparsely vegetated soils (but often shaded), often but not necessarily near water. It is fully winged and capable of flight, and has been observed flying during the daytime. The records for the Boston Harbor ATBI are two specimens that were hand-collected on Thompson Island early in the year by 5^th^ grade students from the Wellesley School District.

#### Bembidion (Lymnaeum) nigropiceum

(Marsham), Invasive

http://species-id.net/wiki/Bembidion_nigropiceum

##### Remarks.

The rediscovery of this species in Massachusetts is of sufficient significance to warrant a separate publication (see Davidson and Rykken, this volume). Hayward (1897) described *Bembidion puritanum* from a few specimens from Massachusetts without further locality, and with no indication of habitat, and it was never seen again. Erwin and Kavanaugh (1980) synonymized Hayward’s species with the European species *Bembidion nigropiceum*. As the species had never been found again in North America, they speculated that it had been introduced but not established. Rediscovery in this survey suggests that the species has been in Massachusetts all along, as it occurs on several of the Boston Harbor Islands (and it also affirms the value of such surveys). We suggest that this species’ flightlessness, small size and apparently very restricted habitat preferences (see Davidson and Rykken, this volume; and Neri and Magrini 2010) resulted in an absence of collecting records for over a century. The biology of this species was unknown (Larochelle and Larivière 2003). The species occurs primarily in a very narrow littoral zone in gravel pushed up at the upper limit of tidal wash. It is short-winged and incapable of flight. The survey collected 82 specimens as follows: Grape Island (one specimen, pitfall, 2008); Rainsford Island (9 specimens, by hand, 2008); and Thompson Island (four specimens, pitfall, 2007; 67 specimens, by hand, 2008; one specimen, by hand, 2010).

#### Brachinus (Neobrachinus) vulcanoides

Erwin

http://species-id.net/wiki/Brachinus_vulcanoides

##### Remarks.

This species seems to be relatively rare in collections and limited to coastal habitats, though nothing seems to be known about its habitat requirements. This species reaches its northern limit in Massachusetts and New Hampshire; Bousquet (2010) does not report any records from further north or east. Published records show a coastal distribution in New Hampshire, Massachusetts, New York, New Jersey, and Florida, thus with a substantial geographic gap between New Jersey and Florida. Lack of collecting effort and difficulty in identification probably underlie this apparent absence. The species of *Brachinus* are difficult to identify at best, but the particular subgroup to which *Brachinus vulcanoides* belongs is notoriously difficult. A relatively modest collecting effort would probably close the distributional gap and perhaps illuminate which ecological requirements limit this species to coastal distribution, as many near relatives are widely distributed. The species is fully winged and presumably capable of flight. Like other *Brachinus*, larvae are presumed to be ectoparasitic on water beetle pupae. All 37 specimens were taken at World’s End.

#### Brachinus (Stenocellus) tantillus

(Dejean), State record

http://species-id.net/wiki/Bradycellus_tantillus

##### Remarks.

The Massachusetts specimen represents a State Record for this species. In New England, this species was known previously from Vermont, Connecticut, Rhode Island and Maine (Bousquet 2010; Sikes 2004; Majka et al. 2011), and in Canada it is not known east of Ontario. Published records from Québec are based on a misidentification (Bousquet 2010). The species occurs on wet clay soils in a variety of wetland habitats. It is fully winged and capable of flight, and is frequently collected at lights. A single specimen was taken.

#### Cymindis (Cymindis) americana

Dejean

http://species-id.net/wiki/Cymindis_americana

##### Remarks.

Specimens from Massachusetts and New Hampshire seem to represent the most northern and eastern records for this species. Québec records are from further west (Larochelle 1975), and it is not recorded from Maine or northeast Canada (Bousquet 2010). The species occurs on dry, sparsely vegetated sandy soils in a variety of open areas (e.g., meadows, pastures). It is wing dimorphic, most individuals brachypterous but some fully winged and capable of flight. A single fully winged specimen was taken.

#### Cymindis (Pinacodera) platicollis

(Say)

http://species-id.net/wiki/Cymindis_platicollis

##### Remarks.

The distributional situation is probably similar to *Cymindis americana*. The species was not recorded from Québec in Larochelle (1975), but Bousquet (2010) mentions Québec records without further locality (though probably closer to Lake Erie and the upper St. Lawrence). It has been recently recorded from Maine (Majka et al. 2011), but without further locality. Thus Massachusetts, New Hampshire and Maine currently are the most northern and eastern records for this arboreal species, and the Maine records may well be from the south. Adults are found for the most part on tree trunks or in the canopy. They are fully winged and capable of flight. Specimens are collected most readily with light traps or by checking tree trunks at night (and especially by baiting tree trunks at night with sugar baits used for attracting moths). Thirteen specimens were taken on four islands and World’s End.

#### Harpalus (Harpalus) rubripes

(Duftschmid), State record, Invasive

http://species-id.net/wiki/Harpalus_rubripes

##### Remarks.

The first report of this European species in North America was Bell and Davidson (1987), who discussed specimens found in New Hampshire and dated as early as 1981. This may have been the point of introduction (or close to it) and it has spread from there both northeast and southwest. It is possible that it was introduced in northeast Canada, as is more typical, and was merely overlooked or confused with the superficially similar *Harpalus affinis* until the presence of *Harpalus rubripes* was made known. But with a number of collectors in the northeast, one would think by now even a misidentified specimen with an earlier year would have been corrected and reported. Either way, the species is now widespread and very common in the northeast, though the Massachusetts specimens are a State Record. By 1993 (Bousquet and Larochelle), it was known from New Hampshire, Rhode Island and Connecticut, and Bousquet (2010) has added Vermont, Maine, Québec, New Brunswick, Nova Scotia and Prince Edward Island. It has spread also considerably to the west, crossing the Hudson River into Ulster County, New York, at least by the year 2000 (NEW STATE RECORD for New York; specimens at Carnegie Museum of Natural History), and during the last decade into the eastern half of Pennsylvania (NEW STATE RECORD for Pennsylvania; specimens from at least Schuylkill, Luzerne and Centre Counties, also in CMNH). The species occurs on dry, sandy, sparsely vegetated soils in a variety of open habitats (e.g., pastures, fields, crops). It is fully winged and capable of flight, and has been taken at lights and in seashore drift. It seems to be widespread among the islands, but not very common. Ten specimens were taken on five islands and World’s End.

#### Laemostenus (Laemostenus) terricola terricola

(Herbst), State record, Invasive

http://species-id.net/wiki/Laemostenus_terricola terricola

##### Remarks.

This is apparently the first record of this species from the United States. *Laemostenus terricola* is known from both coasts of Canada: British Columbia in the northwest (Bousquet and Larochelle 1993), and Québec to Newfoundland in the northeast (Bousquet 2010), with earliest records dating from around 1894. It was not recorded from Québec by Larochelle in 1975, but is recorded by Bousquet in 2010, so presumably it reached Québec in the intervening years (the species is large and usually common once established, so it is not easily overlooked). It is also therefore possibly present in Maine and New Hampshire, though not yet recorded from those states (Bousquet 2010; Majka et al. 2011), unless the specimens from Boston Harbor represent a separate introduction rather than a continuous spreading from northeast to southwest. In spite of its large size and flightlessness (brachypterous (Casale 1988; Larochelle and Larivière 2003)), this species is apparently capable of dispersal. In addition to colonizing both coasts of Canada, it has colonized several sites in India. It is widespread in Europe and Asia from Portugal to the Caucasus, Norway to Romania and Italy. Casale, in his superb monograph on the world’s sphodrines (1988), documented population variation in this species, and indicates that the populations in northeastern Canada came probably from the Iberian Peninsula or from France. Originally from epigean habitats (under stones in open areas, and especially troglophilic and guanophilic in caves and in artificially subterranean environments like nests and burrows), the species is now heavily synanthropic and does very well around human disturbance (gardens, yards, barns, stables, pastures, cellars)(Casale 1988). On the other hand, it seems to have been in the northeast for over one hundred years and, compared with many other introduced carabids, has been relatively slow to spread from its original point or points of colonization. Presumably its habits and habitat render it prone to dispersal by commerce and human disturbance, but once established its large size and flightlessness make local dispersal relatively slow. The three specimens taken on Grape Island (4 October, 2005; 2 July 2008; 14 August, 2008) are the first records from the United States and, of course, a state record for Massachusetts.

One should be aware that there is another, superficially similar *Laemostenus* that is now more or less cosmopolitan, originating from Europe and North Africa, but has reached ports in mid-Atlantic Islands, California, Washington, British Columbia, Peru, Chile, South Africa, Australia, Tasmania and New Zealand. It has not yet been reported from the east coast of North America but should be looked for, and one should not assume that a large *Laemostenus* from the east coast is necessarily *Laemostenus terricola*. This other species, *Laemostenus* (*Laemostenus*) *complanatus* (Dejean), is similarly synanthropic, but is fully winged and presumably capable of flight, and spreads relatively rapidly once colonized.

#### Lebia (Lebia) analis

Dejean

http://species-id.net/wiki/Lebia_analis

##### Remarks.

Specimens from Massachusetts may represent the most eastern and northern records for this species. In Canada it is known only from (presumably southern) Ontario (Bousquet and Larochelle 1993). It is recorded from Vermont and Maine in Bousquet and Larochelle (1993); Vermont but not Maine in Bousquet (2010); and Maine in Majka et al. (2011), but citing specifically Bousquet and Larochelle (1993). We do not know whether the omission of Maine in Bousquet (2010) is an error or a deliberate correction. The species is associated with the chrysomelids *Capraita obsidiana* (Fabricius) and *Disonycha glabrata* Fabricius (Larochelle and Larivière 2003), the carabid larvae presumably being ectoparasitic on the larvae and pupae of the chrysomelids, as is the case with the few *Lebia* species which have been carefully studied. This species is fully winged and capable of flight, and has been taken at lights. One specimen was taken.

#### Lebia (Loxopeza) grandis

Hentz

http://species-id.net/wiki/Lebia_grandis

##### Remarks.

Specimens from Massachusetts and New Hampshire represent the most eastern and (so far) most northeastern records for this species in the northeast. Larochelle (1975) records it from Québec, but only around Lake Ontario and the upper reaches of the St. Lawrence. It is not recorded from Maine or Canada east of Québec (Bousquet 2010; Majka et al. 2011). The larva is known to be an ectoparasite of larvae and pupae of *Leptinotarsa decemlineata* (Say), and probably some other chrysomelids, and is apparently an important predator of the Colorado Potato Beetle (Larochelle and Larivière 2003). Adults are fully winged and capable of flight, and have been taken at lights. One specimen was taken.

#### Lebia (Lebia) viridipennis

Dejean

http://species-id.net/wiki/Lebia_viridipennis

##### Remarks.

Specimens from Massachusetts, New Hampshire and Maine represent the most eastern and northern records for this species. It is recorded from Québec (Bousquet 2010; but not in Larochelle 1975 nor Majka et al. 2011) without further locality, but in Canada not northeast of Québec (Bousquet 2010). It has very recently been recorded from Maine for the first time (Majka et al. 2011). Larvae are presumably ectoparasites on chrysomelids, but hosts seem to be unknown. Adults are fully winged and capable of flight, and have been taken at lights. A single specimen was taken.

#### Scarites (Scarites) subterraneus

Fabricius

http://species-id.net/wiki/Scarites_subterraneus

##### Remarks.

Specimens from Massachusetts and New Hampshire represent the most eastern and northern records for this species to date. It is not reported from Maine (Bousquet 2010), and in Canada it is known only from (presumably southern) Ontario (Bousquet and Larochelle 1993). The species occurs on a variety of soil types in a variety of open habitats, where the subfossorial adults dig burrows. Adults are fully winged and capable of flight, and have been taken at lights. One specimen was taken.

#### Selenophorus hylacis

(Say)

http://species-id.net/wiki/Selenophorus_hylacis

##### Remarks.

Specimens from Massachusetts, New Hampshire and Maine represent the most eastern and northern records for this species to date. It has only recently been reported from Maine (without further locality, Majka et al. 2011) and is not yet known from Canada at all (Bousquet and Larochelle 1993; Bousquet 2010; Majka et al. 2011). The species occurs mainly in or near deciduous forest, is fully winged and capable of flight, and is taken frequently at lights. Two specimens were taken.

#### Stenolophus (Agonoleptus) rotundicollis

(Haldeman)

http://species-id.net/wiki/Stenolophus_rotundicollis

##### Remarks.

Specimens from Massachusetts and Maine represent the most eastern and northeastern records for this species in the northeast. Larochelle (1975) records it from Québec, but only a couple of localities around Lake Ontario, and Bousquet (2010) records it from Vermont. It has not yet been reported from New Hampshire or further to the northeast in Canada, and it has only recently been reported from Maine (Majka et al. 2011). Little is reported about biology, but it seems to be common in lawns and grass. It is fully winged and capable of flight. One specimen was taken.

#### Trichotichnus (Trichotichnus) autumnalis

(Say)

http://species-id.net/wiki/Trichotichnus_autumnalis

##### Remarks.

Specimens from Massachusetts, New Hampshire and Maine represent the most eastern and northern records for this species in the northeast. It is recorded from Québec (Bousquet 2010; but not in Larochelle 1975) without further locality, but in Canada it is not recorded northeast of Québec (Bousquet 2010). It has recently been reported from Maine (Majka et al. 2011), based on recent specimens and a long overlooked record by Harvey and Knight (1897). The species occurs in upland deciduous forests and floodplain forests, is fully winged and capable of flight, and is taken frequently in both lake and seashore drift. A single specimen was taken.

## Discussion

Inherent in any large-scale, multi-taxa inventory are trade-offs between sampling for maximum diversity (involving specialized active techniques for individual taxa) and sampling with maximum efficiency (prioritizing passive “broad spectrum” sampling techniques such as pitfall traps). Our final total of 128 carabid species on the islands, including seven new state records and one new country record, is high relative to other beetle families sampled in the ATBI, but there are undoubtedly many more carabid species to be found in specialized habitats or by using specialized collecting techniques. Our estimate of absolute species richness on the islands is at least 189 species, based on the 47 species of which we collected only one or two specimens. 

Comparisons with the adjacent mainland carabid fauna are necessarily speculative, as there is no comparable recent survey. We chose Sikes’ checklist of Rhode Island Carabidae (2004) as the most complete and comparable reference, recording 306 species. Our comparisons, therefore, of introduced versus native species, and full-winged versus short-winged species, are based on 128 species known so far from the islands and 306 species known so far from Rhode Island, 111 of which are shared between both locations.

Of 128 species on the islands, 14.0% are introduced (18 species), more than twice the percentage of introductions in Rhode Island (17 species, 5.5%), a striking difference. We note that for three of the four introduced species in Boston Harbor that are not recorded in mainland Massachusetts or Rhode Island (*Amara aulica*, *Amara bifrons* and *Laemostenus terricola)* we cannot distinguish whether they are isolated, relatively new introductions limited to the islands, or whether they have spread, undetected, continuously along the coast from other introduction points. Despite the uncertainty regarding the true distribution of these three species, the relatively high proportion of introduced species in Boston Harbor remains noteworthy. The difference may be explained, in part, by the higher proportion of disturbed open dry habitats on the islands, with very little fresh water, as opposed to the greater diversity of older, established habitats (particularly wetlands and fresh water) in Rhode Island. But it is also possible that human traffic and commerce in the islands fosters more frequent introductions, or that these are more likely to become established because of the generally disturbed and depauperate biotic communities present, or a combination of these two. In some cases the islands may even be the point of introduction. This seems undoubtedly the case for the fourth introduced species not known from mainland Massachusetts or Rhode Island, *Bembidion nigropiceum*, as it is not yet known from anywhere else in North America. There is an overall pattern of a high percentage of introduced species recorded in this ATBI relative to the Rhode Island list. For example, the percentage of introduced curculionid beetles in Boston Harbor is 35.4%, compared to 21.4% in Rhode Island (Sikes 2004).

Dispersal ability is an important factor to consider for the colonization of islands in Boston Harbor. The percentages of macropterous (fully winged), wing dimorphic, and brachypterous (short-winged) species were similar for the islands and the mainland. Brachypterous species made up 7.8% of the fauna in both Rhode Island and on the Boston Harbor Islands, while wing dimorphic species made up 11.1% of the total on the mainland versus 14.8% on the islands. The identical rates of brachyptery are surprising, as one would expect isolated islands to have a smaller percentage of short-winged species than the mainland. The percentages of wing dimorphic species cannot be readily interpreted as we did not check the wing status of all individuals of wing dimorphic species. Native short-winged species may have been present already some 15,000 years ago when the islands became isolated post-glaciation. But introduced short-winged species must have arrived on the islands in the last few hundred years. Three of the ten brachypterous species are introductions (*Bembidion nigropiceum*, *Carabus nemoralis* and *Laemostenus terricola*), as are two of the dimorphic species (*Clivina fossor* and *Pterostichus melanarius*). *Carabus nemoralis* was one of the most widespread species, present on seven of the nine islands we sampled intensively. This suggests a high incidence of passive transportation, possibly by human commerce and other human activity. Kotze (2008) attributed high rates of brachyptery in carabid populations on similarly isolated islands in the Baltic Sea, in part, to transportation by drift.

As is commonly the case with carabid surveys, a few species dominated the catch. The six most abundant species (Table 2), numbering over 300 specimens each, are all associated with open, dry areas and/or heavily disturbed areas. Three of them (*Harpalus rufipes*, *Amara bifrons* and *Carabus nemoralis*) are introduced species. *Carabus nemoralis* is brachypterous; *Synuchus impunctatus* is wing dimorphic; the rest are fully winged and capable of flight. The two fully winged introductions are still limited to the northeast; *Harpalus rufipes* is very abundant from Newfoundland to Connecticut, while the nearest recorded mainland populations of *Amara bifrons* are in New Hampshire (no specific locality). The third introduction, *Carabus nemoralis*, is now transcontinental in southern Canada and northern United States, having spread from introduction points near Vancouver and Newfoundland since the earliest recorded specimen (1890, Bousquet 2010). All three native species are common and widespread throughout much of North America, transcontinental in Canada and northern United States, and ubiquitous in New England. Although they now thrive in areas of human disturbance, *Poecilus lucublandus* has always been associated with open areas, and *Pterostichus mutus* and *Synuchus impunctatus* were probably originally inhabitants of open forest and forest edges.

Freshwater is a scarce resource on the Boston Harbor Islands, and the remarkably high species richness on two islands (Calf and Grape; Fig. 2) with freshwater marshes or seeps, attests to the importance of habitat diversity for predicting species richness on an island. Most of the truly hygrophilous species were taken only on Calf and Grape islands, including *Pterostichus corvinus*, *Pterostichus patruelis*, *Pterostichus caudicalis*, several *Agonum* species, most of the *Acupalpus* species, *Pterostichus luctuosus*, and *Agonum melanarium* (the latter two species were also found on World’s End peninsula). Grape Island had the highest species richness of any of the islands, despite its relatively small size, and 26 of the 63 species collected there are associated with fresh water. Calf Island ranked fourth in species richness, with 41 species, in spite of its small size and distance from the mainland. Another hygrophilous species, *Brachinus vulcanoides*, was found only at World’s End, at a single site near a salt marsh. This species may have some salt tolerance, as the species is known so far only from coastal localities.

**Figure:**
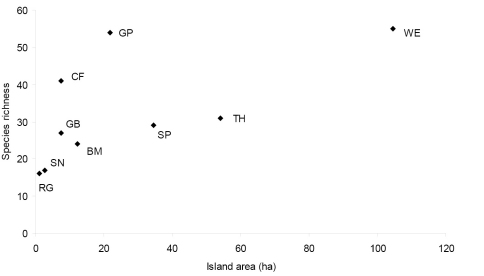
**Figure 2.** Relationship between island area and species richness of carabid beetles in Boston Harbor Islands national park area. Value for species richness has been standardized across all islands to include only one full season of sampling.

The cultural history of an island may also strongly influence species diversity. On Spectacle Island, a recently reclaimed landfill replanted within the last ten years, we collected 29 species and over 1,606 individuals, more carabids than on any other island. This high abundance is due to the dominance of two species, *Amara bifrons* (815) and *Harpalus rufipes* (537), both introduced species and very active colonizers. While a few individuals of *Amara bifrons* were collected on two other islands, on Spectacle Island this species was taken at all five pitfall sites in different habitats. This suggests that Spectacle Island has been invaded relatively recently by this species and is undergoing active and aggressive colonization. The other species, *Harpalus rufipes*, has undoubtedly been in the area much longer, as it has reached seven islands and World’s End peninsula, but over half of the specimens were taken on Spectacle Island. This suggests it has been on Spectacle longer than on the other islands, and it too is actively and aggressively colonizing the island. It may also be that Spectacle has been the jumping off point for invasion of the islands for both species, *Harpalus rufipes* first, and now *Amara bifrons*.

## Conclusion

On islands so variable in cultural history and habitat diversity, and with distances between islands small enough that opportunistic colonization may be commonplace, the classic predictors of species richness proposed by the theory of island biogeography (MacArthur and Wilson 1967)–island size and isolation–may not consistently predict carabid diversity. However, interesting patterns are evident, and we are fortunate to have access to a wealth of published information about carabid natural history and distributions that allows us to interpret some of these patterns. In addition, as our species accounts detail, our work fills in a number of significant geographical (and historical in the case of *Bembidion nigropiceum*) data gaps for the New England carabid fauna. We believe this underscores the value of conducting structured surveys in small accessible natural areas, even in urban areas. While an inventory of the Boston Harbor Islands will not contribute hundreds of new species to science (as has been the case with the ATBI in the Great Smoky Mountain National Park, which had discovered 42 new beetle species as of 2007 (Carlton and Bayles 2007)), it does contribute valuable new information about carabid diversity–and the diversity of over 200 other insect families–in eastern Massachusetts, the most densely human-populated region of New England, and thereby provides relatively easy access to a wealth of educational and scientific opportunities for students, scientists and citizens in the area. After all, naming a species is only the first step in understanding its role in an ecosystem.

## Supplementary Material

XML Treatment for Acupalpus (Acupalpus) hydropicus

XML Treatment for Acupalpus (Acupalpus) nanellus

XML Treatment for Acupalpus (Philodes) rectangulus

XML Treatment for Agonum (Olisares) ferreum

XML Treatment for Agonum (Olisares) punctiforme

XML Treatment for Amara (Curtonotus) aulica

XML Treatment for Amara (Celia) bifrons

XML Treatment for Amara (Bradytus) exarata

XML Treatment for Amara (Amara) ovata

XML Treatment for Apenes lucidulus

XML Treatment for Asaphidion curtum

XML Treatment for Bembidion (Lymnaeum) nigropiceum

XML Treatment for Brachinus (Neobrachinus) vulcanoides

XML Treatment for Brachinus (Stenocellus) tantillus

XML Treatment for Cymindis (Cymindis) americana

XML Treatment for Cymindis (Pinacodera) platicollis

XML Treatment for Harpalus (Harpalus) rubripes

XML Treatment for Laemostenus (Laemostenus) terricola terricola

XML Treatment for Lebia (Lebia) analis

XML Treatment for Lebia (Loxopeza) grandis

XML Treatment for Lebia (Lebia) viridipennis

XML Treatment for Scarites (Scarites) subterraneus

XML Treatment for Selenophorus hylacis

XML Treatment for Stenolophus (Agonoleptus) rotundicollis

XML Treatment for Trichotichnus (Trichotichnus) autumnalis

## References

[B1] Ball GE Bousquet Y (2001) Family 6. Carabidae. In: Arnett RH, Thomas MC, Eds. American Beetles Volume 1: Archostemata, Myxophaga, Adephaga, Polyphaga: Staphyliniformia. CRC Press; Boca Raton, London, New York, Washington DC; 32–132.

[B2] Bell R Buchsbaum RR Roman C Chandler M (2005) Inventory of intertidal marine habitats, Boston Harbor Islands National Park area. Northeastern Naturalist 12: 169-200. 10.1656/1092-6194(2005)12[169:IOIMHB]2.0.CO;2

[B3] Bell RT (1989)*Asaphidion flavipes* Linnaeus in New Hampshire (Coleoptera: Carabidae). The Coleopterists Bulletin 43: 204.

[B4] Bell RT Davidson RL (1987)*Harpalus rubripes* Duftschmid, a European ground beetle new to North America (Coleoptera: Carabidae). The Coleopterists Bulletin 41: 56.

[B5] Bousquet Y (2010) Illustrated Identification Guide to Adults and Larvae of Northeastern North American Ground Beetles (Coleoptera: Carabidae). Pensoft Publishers, Sofia-Moscow, 562 pp.

[B6] Bousquet Y Larochelle A (1993) Catalogue of the Geadephaga (Coleoptera: Trachypachidae, Rhysodidae, Carabidae including Cicindelini) of America north of Mexico. Memoirs of the Entomological Society of Canada 167: 1-397. 10.4039/entm125167fv

[B7] Carlton C Bayles V (2007) Documenting beetle (Arthropoda: Insecta: Coleoptera) diversity in Great Smoky Mountain National park: beyond the halfway point. Southeastern Naturalist 6: 183-192. 10.1656/1528-7092(2007)6[183:DBAICD]2.0.CO;2

[B8] Casale A (1988) Revisione degli Sphodrina (Coleoptera, Carabidae, Sphodrini). Museo regionale di Scienze naturali, Torino, Monografie 5: 1-1024.

[B9] Chao A (1984) Non-parametric estimation of the number of classes in a population. Scandinavian Journal of Statistics 11: 265-270.

[B10] Colwell RK (2009) EstimateS: Statistical estimation of species richness and shared species from samples. Version 8.2. User’s Guide and application. http://purl.oclc.org/estimates.

[B11] Colwell RK Coddington JA (1994) Estimating terrestrial biodiversity through extrapolation. Philosophical Transactions of the Royal Society (Series B) 345: 101-118. 10.1098/rstb.1994.0091 7972351

[B12] Cooper KW (1930) A list of Coleoptera found at Flushing and new to Long Island. Bulletin of the Brooklyn Entomological Society 25: 21-24.

[B13] Davidson RL Langworthy MK (1980) Rediscovery of *Asaphidion flavipes* Linnaeus (Coleoptera: Carabidae) in Long Island, New York. The Coleopterists Bulletin 34: 280.

[B14] Davidson RL Rykken J (2011) Rediscovery of *Bembidion* (*Lymnaeum*) *nigropiceum* (Marsham) (Coleoptera: Carabidae: Bembidiini) in Massachusetts, with remarks on biology and habitat. ZooKeys, this volume. 10.3897/zookeys.147.2105 PMC3286246 22379389

[B15] Elliman T (2005) Vascular flora and plant communities of the Boston Harbor Islands. Northeastern Naturalist 12: 49-74. 10.1656/1092-6194(2005)12[49:VFAPCO]2.0.CO;2

[B16] Erwin TL Kavanaugh DH (1980) On the identity of *Bembidion puritanum* Hayward (Coleoptera: Carabidae: Bembidiini). The Coleopterists Bulletin 34: 241-242.

[B17] Farrell BD (2005) From agronomics to international relations: building an online encyclopedia of life in the Dominican Republic. Revista: Harvard Review of Latin America, Fall 2004/Winter 2005: 6–9.

[B18] Harvey FL Knight OW (1897) Insects collected at Jackman, Maine. Psyche 8: 77-79.

[B19] Hayward R (1897) On the species of *Bembidium* of America north of Mexico. Transactions of the Entomological Society of America 24: 32-143.

[B20] Hieke F (2000) Revision einiger Gruppen und neue Arten der Gattung *Amara* Bonelli, 1810 (Coleoptera: Carabidae). Annales Historico-naturales Musei Nationalis Hungarici 92: 41-143.

[B21] Janzen DH Hallwachs W (1994) All Taxa Biodiversity Inventory (ATBI) of Terrestrial Systems: A Generic Protocol for Preparing Wildland Biodiversity for Non-Damaging Use. Report of a National Science Foundation Workshop, 16–18 April 1993, Philadelphia, PA, 132 pp.

[B22] Kales D (2007) The Boston Harbor Islands: a history of an urban wilderness. The History Press, Charleston, SC, 158 pp.

[B23] Kotze J (2008) The occurrence and distribution of carabid beetles (Carabidae) on islands in the Baltic Sea: a review. Journal of Insect Conservation 12: 265-276. 10.1007/s10841-008-9147-4

[B24] Krinsky WL (1981) First report of *Asaphidion flavipes* Linnaeus (Coleoptera: Carabidae) in the United States outside of Long Island, New York, and some biological observations. The Coleopterists Bulletin 35: 477-478.

[B25] Larochelle A (1975) Les Carabidae du Québec et du Labrador. Département de Biologie du Collège Bourget, Rigaud, Bulletin 1: 1-255.

[B26] Larochelle A Larivière MC (2003) A Natural History of the Ground-beetles (Coleoptera: Carabidae) of America North of Mexico. Pensoft Publishers, Sofia-Moscow, 583 pp.

[B27] Lindroth CH (1961) The ground-beetles (Carabidae, excl. Cicindelinae) of Canada and Alaska. Part 2. Opuscula Entomologica Supplementum 20: 1-200.

[B28] Lindroth CH (1963) The ground-beetles (Carabidae, excl. Cicindelinae) of Canada and Alaska. Part 3. Opuscula Entomologica Supplementum 24: 201-408.

[B29] Lindroth CH (1966) The ground-beetles (Carabidae, excl. Cicindelinae) of Canada and Alaska. Part 4. Opuscula Entomologica Supplementum 29: 409-648.

[B30] Lindroth CH (1968) The ground-beetles (Carabidae, excl. Cicindelinae) of Canada and Alaska. Part 5. Opuscula Entomologica Supplementum 33: 649-944.

[B31] Lindroth CH (1969a) The ground-beetles (Carabidae, excl. Cicindelinae) of Canada and Alaska. Part 6. Opuscula Entomologica Supplementum 34: 945-1192.

[B32] Lindroth CH (1969b) The ground-beetles (Carabidae, excl. Cicindelinae) of Canada and Alaska. Part 1. Opuscula Entomologica Supplementum 35: i-xlviii.

[B33] MacArthur RH Wilson EO (1967) The Theory of Island Biogeography. Princeton University Press, Princeton, NJ, 203 pp.

[B34] Majka C Chandler DS Donahue CP (2011) Checklist of the Beetles of Maine. Empty Mirrors Press, Halifax, Nova Scotia, Canada, 328 pp.

[B35] Mello MJ (2005) Inventory of macrolepidoptera and other insects in the Boston harbor Islands national park area. Northeastern Naturalist 12: 169-200. 10.1656/1092-6194(2005)12[99:IOMAOI]2.0.CO;2

[B36] National Park Service (2002) Boston Harbor Islands: a national park area. General Management Plan, Boston Support Office of the Northeast Region, Boston, MA, 192 pp.

[B37] Neri P Magrini P (2010) Note concernenti i *Bembidion* appartenenti al sottogenere *Lymnaeum* Stephens, 1828 (Insecta Coleoptera Carabidae). Quaderno di Studi e Notizie di Storia Naturale della Romagna 31: 135-154.

[B38] Nichols B Langdon KR (2007) The Smokies All Taxa Biodiversity Inventory: history and progress. Southeastern Naturalist 6: 27-34. 10.1656/1528-7092(2007)6[27:TSATBI]2.0.CO;2

[B39] Sikes DS (2004) The Beetle Fauna of Rhode Island: an Annotated Checklist. Volume 3 of The Biota of Rhode Island. The Rhode Island Natural History Survey, Kingston, Rhode Island, 296 pp.

